# Mapping biocultural diversity: An integrative spatial approach to inform conservation and management decisions

**DOI:** 10.1007/s13280-026-02370-6

**Published:** 2026-03-14

**Authors:** Irene Otamendi-Urroz, Juan Miguel Requena-Mullor, Cristina Quintas-Soriano, Graeme S. Cumming, Antonio J. Castro

**Affiliations:** 1 Social-Ecological Research Laboratory, Biology and Geology Department, Andalusian Center for Global Change - Hermelindo Castro (ENGLOBA), University of Almeria, Carretera Sacramento S/N, 04120 La Cañada de San Urbano, Almería, Spain; 2 FRACTAL Collective, Madrid, Spain; 3 Oceans Graduate School, UWA Oceans Institute, University of Western Australia, 35 Stirling Highway, Perth, 6009 Australia

**Keywords:** Biodiversity, Culture, Hotspots, Indicators, Social-ecological, Spatial analysis

## Abstract

Effective conservation and sustainable management require metrics that capture the coupled dynamics of biological and cultural systems. Quantifying and mapping this biocultural diversity remains difficult due to its multidimensional and context-dependent nature. This study introduces the Biocultural Index for Refugia Exploration (BIRE), an integrative spatial approach combining biological and socio-cultural variables to map biocultural diversity. Using publicly available datasets, BIRE applies a percentile-based scoring system to identify biocultural diversity hotspots at the municipal scale. Tested in two contrasting Spanish regions (Almería and Navarra), BIRE revealed distinct spatial patterns, with higher values representing hotspots in rural and mountainous municipalities and lower values (coldspots) in urbanized areas. The index proved robust, flexible, and replicable across diverse social-ecological contexts. BIRE offers a policy-relevant framework linking scientific evidence with decision-making processes. In doing so, BIRE can guide targeted interventions in sustainable management, conservation, and planning, supporting decision-makers in identifying and prioritizing key areas.

**Supplementary Information:**

The online version contains supplementary material available at https://doi.org/10.1007/s13280-026-02370-6.

## Introduction

As anthropogenic pressures on ecosystems intensify, there is increasing acknowledgment that these issues transcend strictly ecological, social, or economic domains. Addressing global sustainability challenges such as biodiversity loss, climate change, resource depletion, and social inequality requires not only safeguarding natural systems but also ensuring human well-being while recognizing the complex interconnections between cultural practices and ecosystems (Rapport and Maffi 2010).

The concept of ‘biocultural diversity’ emerged to holistically address how human societies and natural ecosystems are interconnected and influence one another (Pretty et al. 2009). It emphasizes that human-nature relationships are dynamic, context-specific, and shaped by distinct histories, knowledge systems, beliefs, and practices, which manifest spatially in landscapes and are closely linked to local biodiversity (Cocks 2006; Agnoletti and Rotherham 2015; Bridgewater and Rotherham 2019; Ingold 2021). Within this perspective, particular attention has been given to landscapes of high biocultural value—often referred to as biocultural refugia—where both biological and cultural diversity have been maintained over time, frequently due to traditional practices, communal governance, or relative isolation from intensive development (Loh and Harmon 2005). These refugia contribute to the resilience of social-ecological systems by preserving adaptive strategies and acting as ‘libraries’ of traditional knowledge (Barthel et al. 2013; Sõukand and Pieroni 2019).

Since its initial articulation by Maffi (2007), ‘biocultural diversity’ has been defined and redefined multiple times (Bridgewater and Rotherham 2019). In this study, we adopt the operational definition proposed by Loh and Harmon (2005), which states that biocultural diversity includes biological diversity at all its levels, from genes to populations, species, and ecosystems; cultural diversity spanning from individual ideas to entire cultures; and all the interactions among them. This definition provides concrete examples of what can be considered biocultural diversity at different scales and offers the level of specificity needed to translate a complex concept into quantifiable variables, making it particularly suitable for operationalization in empirical and spatial analyses (Hanspach et al. 2020).

Operationalization requires frameworks, methodologies, and metrics to capture biocultural diversity’s multidimensional nature. For instance, the conceptual framework proposed by Elands et al. (2019) categorize biocultural diversity into three main dimensions: (1) materialized (i.e., tangible biophysical expressions of biocultural diversity); (2) lived (i.e., everyday interactions between people and nature mediated by our senses and minds); and (3) stewardship (i.e., motivations, attitudes, and actions promoting awareness and the active engagement in caring for biocultural diversity). However, the development of multidimensional and spatial metrics for biocultural diversity quantification remains limited (Correia et al. 2021), even though these metrics are widely recognized as essential tools in ecology, especially for assessing ecosystem services (Sherrouse et al. 2011). One of the earliest and most influential attempts to quantitatively assess and spatially map biocultural diversity was Loh and Harmon’s (2005) global index of biocultural diversity, which relied on a relatively small set of variables and a broad spatial resolution. Subsequent studies (Gottero and Cassatella 2017; Dobrovodská et al. 2019; Gonçalves et al. 2021; Echeverri et al. 2025) have advanced the field by developing refined methods to better capture biocultural diversity at regional and local scales, and to integrate both tangible and intangible aspects. Spatially explicit participatory mapping approaches (Pert et al. 2015; Wengerd and Gilmore 2022) have also provided rich, fine-scale insights into local biocultural values, but often required substantial fieldwork, time, and resources to collect site-specific data. More recently, new initiatives have emerged aimed at adapting ecological indicators (e.g., Shannon and Simpson diversity indices) as proxies for biocultural diversity (Mateus-Aguilar et al. 2025), which offer innovative analytical pathways but use metrics not originally designed to capture the full holistic nature of the concept.

While these advances mark substantial progress, several pressing issues remain unresolved, particularly how to effectively integrate diverse biological and cultural variables within a single analytical framework. Addressing this gap calls for complementary approaches that rely on publicly available spatial datasets to ensure cost-effective and replicable applications, while operationalizing biocultural diversity in a genuinely multidimensional manner. Such approaches should be conceptually organized around the materialized, lived, and stewardship dimensions of biocultural diversity (Elands et al. 2019). This framework can then be applied across regional and local case studies encompassing diverse social-ecological systems along the rural-urban gradient. At the same time, there is a need to translate the concept of biocultural diversity into practical and actionable tools for managers, planners, and policymakers (Bridgewater and Rotherham 2019; Lukawiecki et al. 2022), including methods to identify these biocultural refugia and to support the adaptive strategies for sustainability and resilience embedded within them (Barthel et al. 2013; Sõukand and Pieroni 2019).

In this study, we address some of these challenges by introducing and developing the Biocultural Index for Refugia Exploration (BIRE). Designed as a new practical and scalable tool, BIRE also serves as a testing ground for refining and advancing more robust, coherent, and transferable metrics of biocultural diversity, thereby supporting informed decision-making in conservation, management, and planning. To illustrate its utility, our main objectives were to (1) evaluate the robustness and internal consistency of the index; (2) map BIRE-based hotspots and coldspots; and (3) describe and interpret the spatial distribution of biocultural diversity as measured by BIRE. To achieve these objectives, we applied our methodology to two contrasting regions of Spain: Navarra in the north, and Almería in the south. These regions represent markedly different social-ecological contexts, offering an ideal testing ground to evaluate whether the index performs effectively across diverse environmental and cultural settings. Beyond the present study, the index can be used not only to identify biocultural diversity hotspots, but also to support deeper investigations into whether these areas function as biocultural refugia.

## Materials and methods

**Fig. 1:**
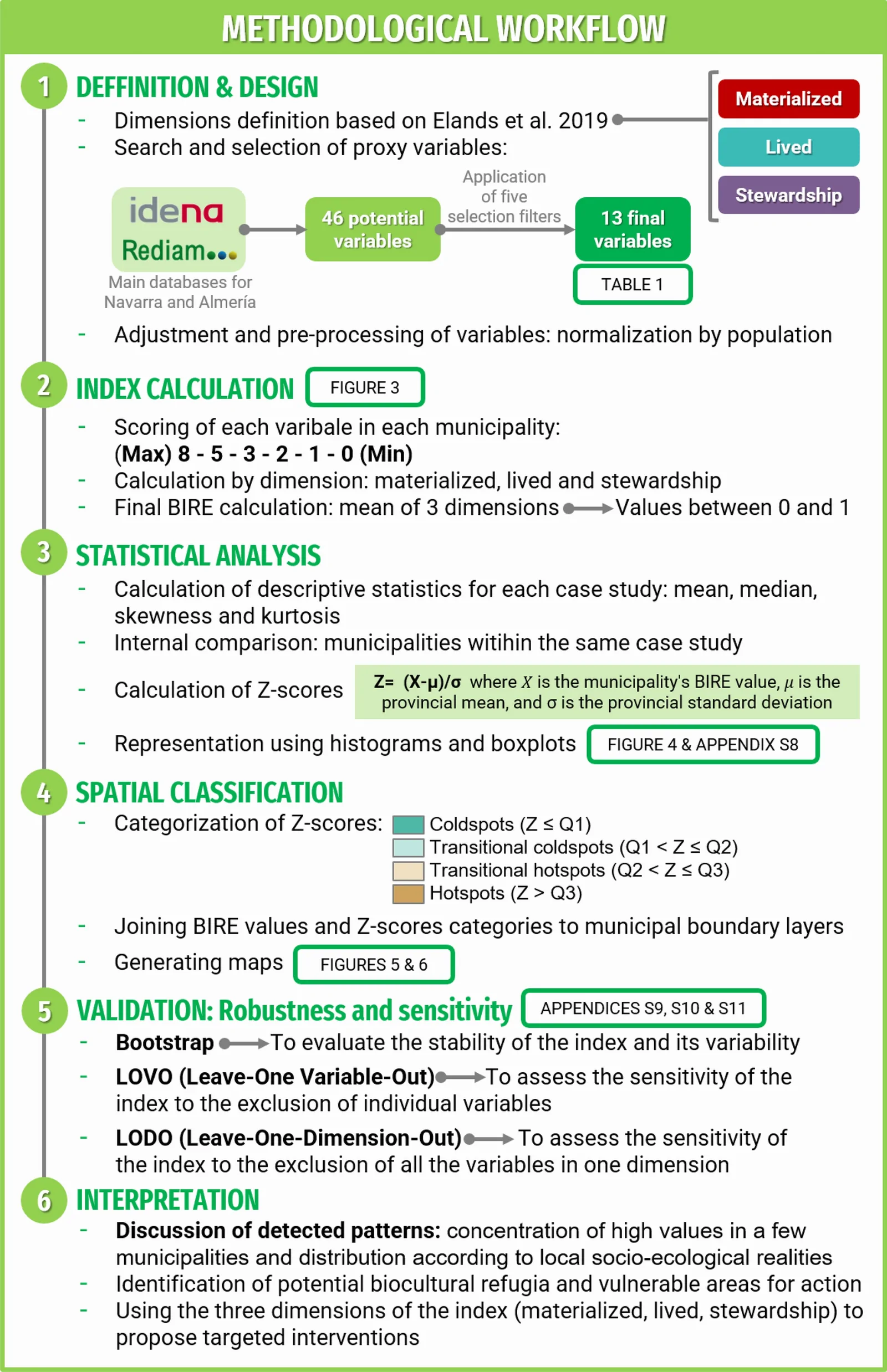
Methodological workflow for the Biocultural Index for Refugia Exploration (BIRE)

We designed a multi-step methodological workflow that integrates data collection, index construction, statistical treatment, spatial analysis, and explanatory analysis. This workflow ensures transparency and reproducibility by linking each stage of the process. Figure 1 provides an overview of the six main steps.

### Case studies

**Fig. 2:**
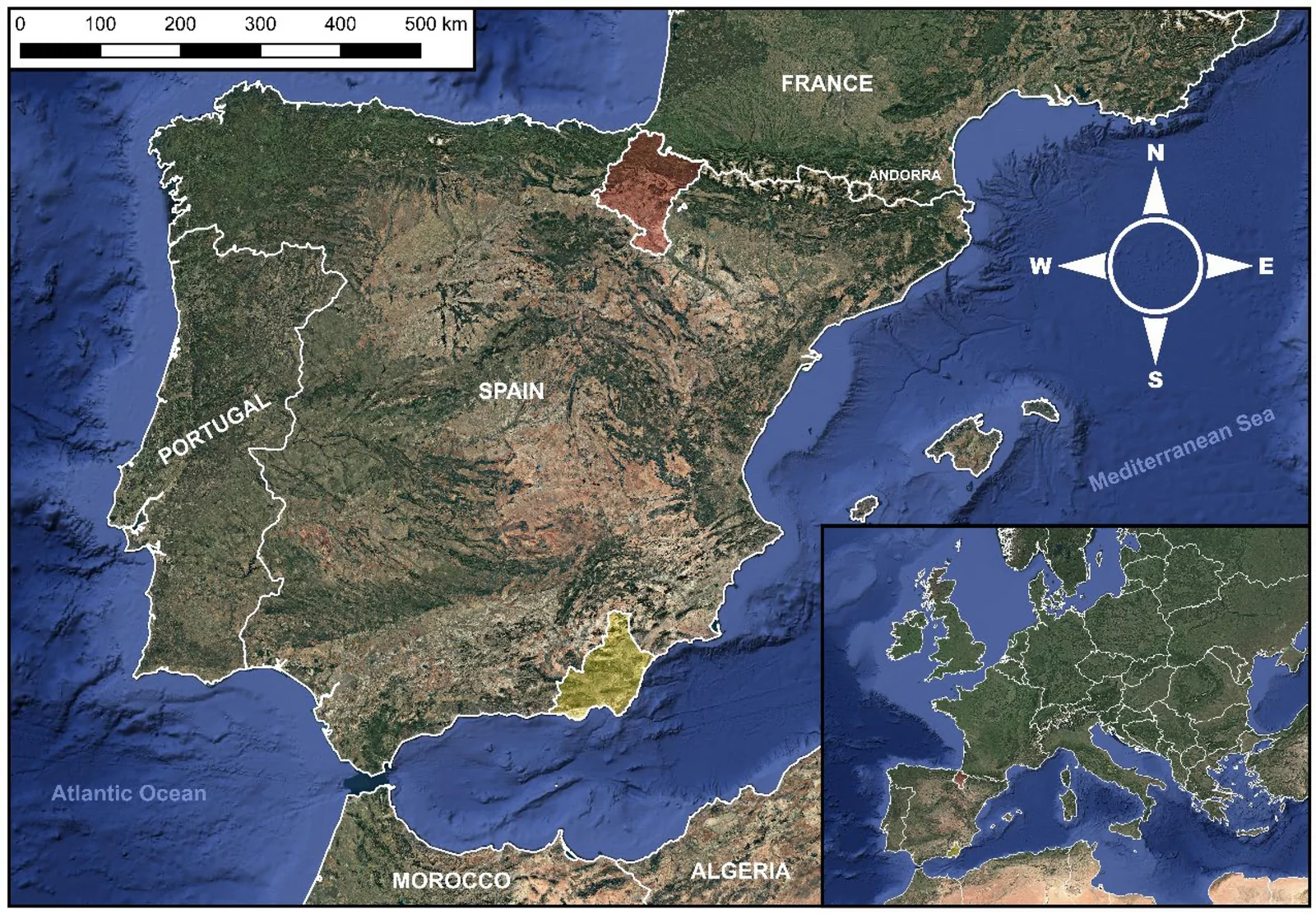
Geographical location of case studies. Navarra is outlined in red and Almería in yellow

We selected two different regions in Spain: the province of Almería (southeast) and the autonomous community of Navarra (north) (Fig. 2). These regions were chosen based on the availability of comprehensive and spatially explicit datasets and on the need to test the index robustness and adaptability across distinct contexts, capturing the climatic, geographical, and socio-economic differences between northern and southern Spain. Despite their differences, both regions share a national legal and regulatory framework, as well as historical ties, which help control external influences.

Almería is located in the autonomous community of Andalusia and bordered by the Mediterranean Sea to the south and east. The province encompasses a wide range of landscapes including Mediterranean shrublands, desert, dry forests, high-mountain areas, and coastal plains (Otamendi-Urroz et al. 2023), harbouring high levels of plant endemism and specialized fauna adapted to water-limited environments (Matamala 2007). The subdesertic–Mediterranean subclimate predominates with low annual precipitation (below 300 mm) and high summer temperatures (reaching 40 ºC), while cooler, wetter conditions (1000 mm precipitation) are present in high-elevation areas (Gómez-Zotano et al. 2015). Covering 8774 km^2^, Almería includes 102 municipalities and a population of 730 430 inhabitants (INE 2022). Its economy is mainly driven by: (1) intensive agriculture (320.48 km^2^ of greenhouses) (Regional Government of Andalusia 2019) and (2) tourism, mainly beach-centered (Quintas-Soriano et al. 2016). From a biocultural perspective, inland areas retain traditional agro-pastoral landscapes (e.g., olive and almond groves, terraced fields, and extensive livestock grazing) adapted to aridity, while coastal zones historically linked to marine ecosystems and small-scale fisheries have been increasingly transformed by greenhouse industry, tourism, and urbanization (Quintas-Soriano et al. 2016). The province’s cultural identity reflects a strong Arab legacy combined with predominantly Christian traditions (e.g., Eastern processions) and a broader Andalusian cultural identity (e.g., gastronomy, music, and livelihoods) (Martínez-López 2006). Ongoing land abandonment, rural depopulation, water scarcity, and coastal intensification are contributing to the erosion of Almería’s traditional biocultural landscapes (Quintas-Soriano et al. 2016, 2023).

The autonomous community of Navarra is located in northern Spain, bordering France to the north. Navarra features diverse landscapes transitioning from the Pyrenees Mountains with high peaks and dense forests, to the fertile plains of the Ebro River Valley in the south (Gobierno de Navarra 2021). Climate transitions from Atlantic in the north (900–2500 mm precipitation, mild winters at 6–12 °C and cool summers between 11 and 26 °C); to Mediterranean in the south (400–600 mm precipitation and 35–40 °C maximum summer temperatures) (Servicio de Meteorología de Navarra 2024). In the northeastern Pyrenees, an Alpine climate prevails, with heavy snowfall in winter and temperatures frequently below zero (Servicio de Meteorología de Navarra 2024). This topographic and climatic heterogeneity supports a high diversity of habitats and a great variety of species adapted to altitudinal and climatic gradients (Pejenaute-Goñi 2002; Peralta et al. 2018). Covering 10 391 km^2^, Navarra comprises 272 municipalities and 668 858 inhabitants (INE 2022). Its economy is mainly driven by: (1) agriculture and livestock farming; (2) automotive industry and renewable energy industries; and (3) tourism, linked to Pamplona city and nature-based outdoor activities (Pejenaute-Goñi 2002). From a biocultural perspective, Navarra is characterized by a strong regional identity rooted in the Basque language (Euskera), a long history as an independent kingdom (Reyno de Navarra), and enduring communal land tenure systems and local governance institutions (Pejenaute-Goñi 2002). These factors have fostered close connections between people and landscapes through practices such as communal grazing, transhumant pastoralism, forest use traditions, seasonal festivals linked to agrarian cycles, and rural cultural expressions (e.g., Basque rural sports 'herri kirolak') (Pejenaute-Goñi 2002). In recent decades, demographic aging, rural depopulation, urban expansion, and agricultural modernization have increasingly challenged the continuity of these connections (Labiano-Mangado 2021; Albizua et al. 2024).

Although these two contrasting regions were selected as case studies, municipalities within each region constitute the unit of analysis. All variables, index values, and statistical classifications were calculated and interpreted at the municipal level. Consequently, comparisons of BIRE values, categories (coldspots to hotspots), and outliers are valid only among municipalities within the same region. Navarra and Almería are therefore not directly compared with each other; rather, they are used as contrasting social-ecological contexts to test the robustness, scalability, and applicability of the index under different environmental, cultural, and data availability conditions.

### Data compilation and variable selection

We conducted an extensive search of spatial information in regional environmental and statistical databases, including IDENA (Navarra’s Geographic Information System;^1^) and REDIAM (Andalusian Environmental Information Network;^2^), alongside national spatial and statistical resources and supplementary web-based data (Appendix S1).

We searched for variables that could serve as municipal level proxies for the three dimensions of biocultural diversity (i.e., materialized, lived, and stewardship) (Elands et al. 2019). To reduce the large number of potential variables identified, we applied five filters. First, we prioritized conceptually relevant variables available in both study areas. Second, we excluded those lacking full spatial coverage. Third, we used Principal Component Analysis (PCA) and a Spearman correlation threshold; 0.8 in R to remove redundant variables, balancing collinearity reduction with the need to retain distinct information. Fourth, when several variables represented similar phenomena (e.g., area versus number of Habitats of Communitarian Interest), we selected the most straightforward based on our expertise. Finally, we favored variables with clearer spatial interpretability to allow meaningful analysis of their distribution and significance.

In total we selected 13 variables (Table 1) as proxies for biocultural diversity (our response or ‘Y’ variables). Of these, 10 were used to construct our biocultural diversity index for Almería and 11 for Navarra (Appendix S2). In the context of the materialized dimension, we chose variables such as species richness or landscape diversity. For the lived dimension, we gathered variables related to agricultural practices, livestock practices or cultural traditions, among others. Lastly, for the stewardship dimension, we selected variables reflecting communal property and protection norms.

**Table 1:** Proxies for biocultural diversity. In the 'variable' column, superscripts are used as follows: a = Variables used for Almería; n = Variables used for Navarra. Links to information sources are available in the References section

Dimension	Variable	Description	Source
MATERIALIZED	Species richness^a,n^	Number of different terrestrial species (non-vascular flora, vascular flora, invertebrates, and vertebrates) present in the grid cells (10 × 10 km) corresponding to each municipality	MTERD (2017)
	Landscape types^a,n^	Number of different landscape types appearing simultaneously in one municipality	REDIAM (2023), IDENA (2023)
	Habitats of Communitarian Interest^a,n^	Total number of Habitats of Communitarian Interest defined by the European Union Habitats Directive occurring simultaneously in the same municipality	MTERD (2018)
LIVED	Toponyms^a,n^	Sum of the total number of 'toponyms' registered for the area inside each municipality (a 'toponym' is a place name, especially one derived from a topographical feature)	REDIAM (2023), IDENA (2023)
	Festivities and traditions^a^	Number of different festivities and traditions reported in each municipality	Diputación de Almería (n.d.), IAPH (2024)
	Transhumance^a,n^	Sum of the length (in meters) of all the different transhumance routes present in each municipality	REDIAM (2023), IDENA (2023)
	Hunting^a,n^	Sum of the area (km^2^) in which major or minor hunting are allowed	REDIAM (2023), IDENA (2023)
	Fishing^n^	Sum of the river length (meters) where fishing is allowed in each municipality	IDENA (2023)
	Logging^n^	Area (km^2^) in which logging is allowed and practiced in each municipality	IDENA (2023), IDENA (2023)
	Traditional agriculture^a,n^	Area (km^2^) under open-air agricultural land use, used here as a proxy for traditional agricultural landscapes and excluding greenhouse-based agriculture, in each municipality	CNIG (2014)
STEWARDSHIP	Protected area^a,n^	Area (km^2^) under any protection figures in each municipality	REDIAM (2023), IDENA (2023)
	Communal area^a,n^	Area (km^2^) managed by local entities in communal regime in each municipality	MTERD (2023)

Proxy variables were assessed at the municipality level, as it constitutes the smallest administrative boundary for land management in Spain (Quintas-Soriano et al. 2019). This final set of variables (Appendix S2) was normalized by the total population of each municipality (INE 2022) to ensure comparability across municipalities within each case study. The difference in the number of variables between both case studies was due to data availability (e.g., while comprehensive information on local festivities was available for all municipalities in Almería, this was not the case for Navarra based on online sources).

### Biocultural diversity index

**Fig. 3:**
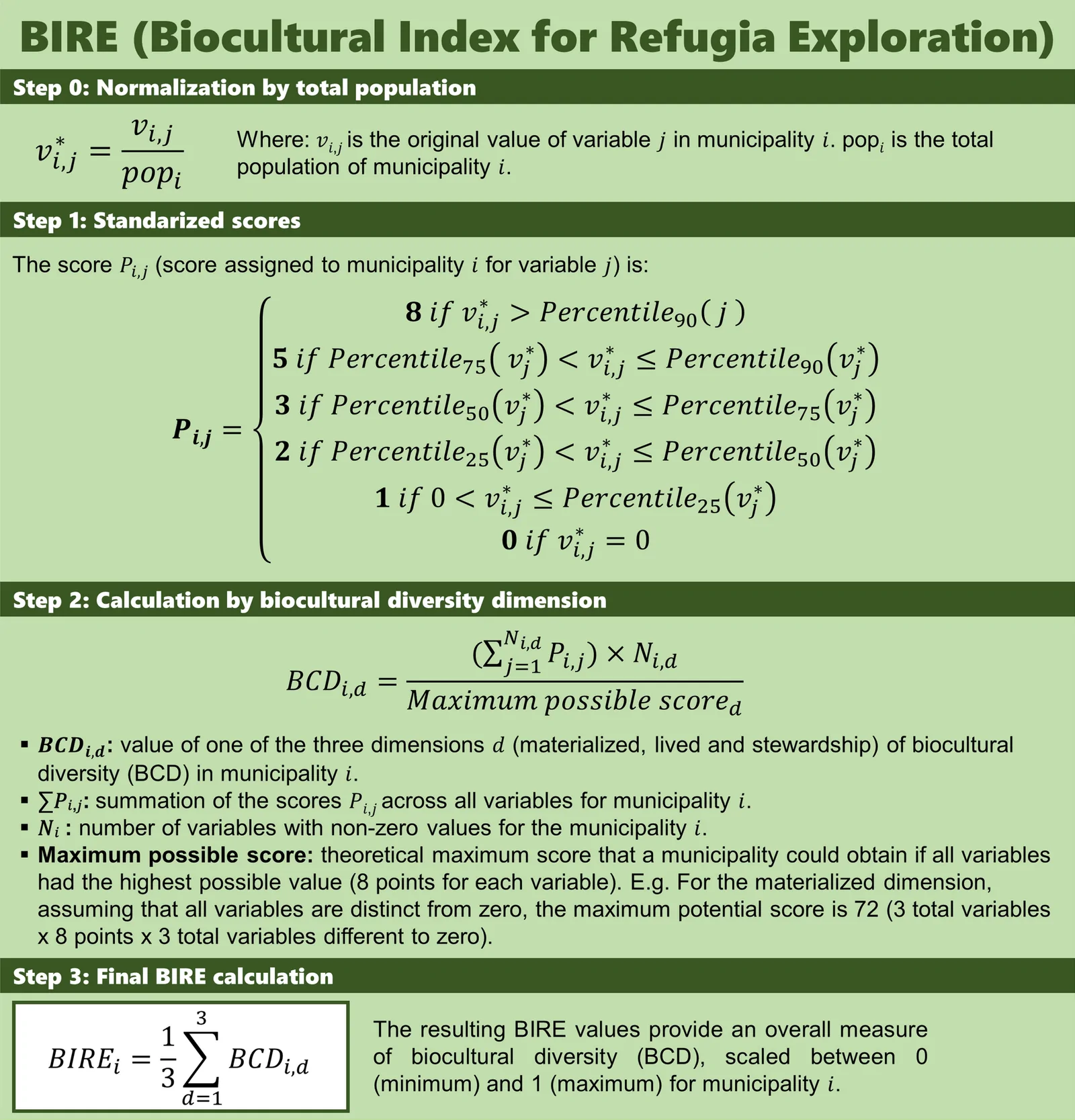
Steps for calculating the Biocultural Index for Refugia Exploration (BIRE)

The Biocultural Index for Refugia Exploration (BIRE) was fully computed in R (version 4.3.1) following a three-step process (Fig. 3) (see Appendix S6 for an illustrative example). First, according to the value of each variable in each municipality, a score (0, 1, 2, 3, 5, or 8) was assigned based on the interval ranges defined by the 25^th^, 50^th^, 75^th^, and 90^th^ percentiles (Step 1 in Fig. 3). This approach accounted for differences in the range of the original variables.

Next, the scores were separately summed for the materialized, lived, and stewardship biocultural dimensions and multiplied by the number of variables in each dimension with a value greater than zero in the given municipality. This multiplication accounted for cases where distinct municipalities had the same summation but different numbers of biocultural diversity variables with nonzero values, reflecting variations in biocultural richness. To ensure interpretability, the summed scores were normalized by dividing by the maximum possible score for each dimension (Appendix S6). This resulted in three values, each ranging from 0 to 1, representing the normalized scores for each dimension (Step 2 in Fig. 3).

The final step involved averaging the three normalized scores to obtain a unified/combined measure of biocultural diversity (Step 3 in Fig. 3). The resulting BIRE values provide an overall index for biocultural diversity, scaled between 0 (minimum) and 1 (maximum). A higher BIRE value indicates that a municipality has consistently high scores across multiple variables, meaning that its proxies for biocultural diversity are relatively abundant and well distributed across the three dimensions considered. Conversely, a lower BIRE value suggests fewer or less prominent biocultural diversity components in the municipality. It is important to highlight that, since all variables were first normalized by population, these variables reflect biocultural components per capita, enabling meaningful comparisons between municipalities of the same region regardless of their size. Additionally, municipalities can exhibit variations in different dimensions of biocultural diversity (i.e., materialized, lived, and stewardship), meaning that two municipalities with similar overall BIRE values may have different compositions of biocultural components (Appendix S7).

Once the index was constructed and computed, robustness and sensitivity analyses were conducted. First, a Leave-One-Variable-Out (LOVO) approach was applied in R (Appendix S10), where each variable was iteratively excluded and the index recalculated to evaluate its influence on the final results. Additionally, a 'Leave-One-Dimension-Out' (LODO) analysis was performed, in which entire groups of explanatory variables (i.e., materialized, lived, or stewardship) were excluded one at a time (Appendix S10). This allowed us to assess whether all variables and dimensions were necessary for capturing biocultural diversity or if some groups introduced redundancy or systematic bias not attributable to methodological choices. Finally, bootstrap simulations (Tibshirani and Efron 1993) were performed (Appendix S9). By repeatedly resampling the dataset, these simulations generated confidence intervals, providing a measure of variability and assessing the stability of the index under different data samples.

To describe the distribution of BIRE values in each case study, the index (BIRE_i_) was calculated for all municipalities in each case study and then, descriptive statistics were computed in R, including mean, median, standard deviation, skewness, and kurtosis (Table 2). These metrics provide insights into the central tendency, dispersion, and shape of the distribution. Additionally, histograms (Appendix S8) were generated to visualize the distribution of BIRE values across municipalities in both regions.

**Table 2:** Statistical properties of BIRE in Almería and Navarra

Statistical properties	Almería	Navarra
Mean	0.35	0.31
Median	0.32	0.27
Standard deviation	0.20	0.18
Skewness	0.58	0.86
Kurtosis	2.65	3.35

We standardized the index by calculating Z-scores, which expressed how much a municipality’s index deviates from the provincial mean. This transformation allows for a relative comparison of values across municipalities, independent of the overall scale of the index. The Z-score was calculated in R as follows: $Z = \frac{X - \mu }{\sigma }$ where *X* is the municipality's BIRE value, μ is the provincial mean, and σ is the provincial standard deviation. Then, municipalities were categorized into four groups using a quartile-based classification: coldspots (Z-score ≤ Q1), transitional coldspots (Q1 < Z-score ≤ Q2), transitional hotspots (Q2 < Z-score ≤ Q3), and hotspots (Z-score > Q3), where Q represents the value of the first, second, and third quartiles dividing the dataset into four equal parts. The classification was applied independently for each region, meaning that comparisons of categories are only valid between municipalities within the same case study. This approach provided a straightforward and intuitive way to highlight areas with relatively high or low biocultural diversity according to BIRE values. Boxplots were also used to examine the spread of standardized values and detect potential outliers within each region.

To complement the analysis, we also applied a variance partitioning approach in R to disentangle the relative contributions of climatic, geographic/land-use, and demographic variables (Appendix S3) to the variability of BIRE values; detailed methods and results are provided in Appendix S11.

### Mapping exercise

We mapped all variables (corrected per municipality population) and visualized their spatial distribution in Navarra (Appendix S4) and Almería (Appendix S5) using QGIS 3.32.2^3^. Z-scores were calculated, the quartile-based classification was applied, and the results were subsequently joined from an Excel file to the municipal boundary layer in QGIS, where each category was assigned a distinct color scheme. We created final maps depicting: (1) BIRE general values per each municipality and its coldspots and hotspots (Fig. 5); and (2) BIRE specific values for materialized, lived, and stewardship dimensions separately (Fig. 6). These maps allowed comparisons between municipalities within the same province and the identification of potential biocultural refugia and priority areas for biocultural diversity conservation. In Navarra, ‘facerías’ (i.e., shared-use lands collectively managed by various municipalities), marked in gray on the maps, were not analyzed as they do not constitute municipalities and lack a population census, making it impossible to normalize the variables by population.

## Results

### Sensitivity, robustness, and summary statistics of BIRE in the case studies

Sensitivity analyses confirmed the overall robustness of BIRE, showing that no single variable disproportionately affected the municipal rankings. The LOVO analysis (Appendix S10) revealed only minor changes in index values, with high correlations and low root mean square error (RMSE) values. The exclusion of stewardship variables such as protected and communal areas produced larger deviations, indicating they capture unique and non-redundant aspects of biocultural diversity. Consequently, this demonstrates BIRE's capacity to identify and weight impactful elements, even when they are infrequent and belong to a dimension represented by only two variables.

Parallel LODO analysis (Appendix S10) demonstrated that each dimension provides essential, non-redundant information. The removal of any single dimension diminished the index's capacity to capture biocultural diversity; with the strongest effect for stewardship, whose removal reduced correlations and increased change rates. This finding powerfully emphasizes that stewardship captures distinctive and non-substitutable aspects of biocultural diversity.

Bootstrap simulations (Appendix S9) confirmed numerical stability, with mean estimates of 0.571 (95% CI 0.542–0.592) in Almería and 0.479 (95% CI 0.462–0.493) in Navarra, both with negligible bias and low standard errors (0.013 and 0.008, respectively). These narrow confidence intervals demonstrate that the index is robust and not overly sensitive to sampling variability.

BIRE descriptive statistics for both case studies are shown in Table 2. While the absolute index values are not directly comparable due to differences in the variables used for each case study, their statistical distributions can be contrasted. On average, municipalities in Almería exhibited slightly higher BIRE values than those in Navarra, as reflected by both the mean and median. The distributions in both regions showed moderate positive skewness, suggesting that a majority of municipalities have relatively low to moderate BIRE values, with fewer municipalities exhibiting high values. Additionally, kurtosis values indicated that the distributions were slightly more peaked than a normal distribution, particularly in Navarra. Histograms in Appendix S8 illustrate the empirical distribution within each case study.

In both case studies, most municipalities had BIRE values that were relatively balanced around the provincial mean. However, Navarra exhibited a slightly higher skewness (Table 2), with several municipalities having higher-than-expected BIRE values. In contrast, Almería presented a more balanced distribution, with fewer extreme values. These differences suggested that biocultural diversity components might be more evenly distributed among municipalities in Almería, while Navarra had greater variability and a few municipalities with notably high BIRE values.

### BIRE and hotspots mapping

**Fig. 4:**
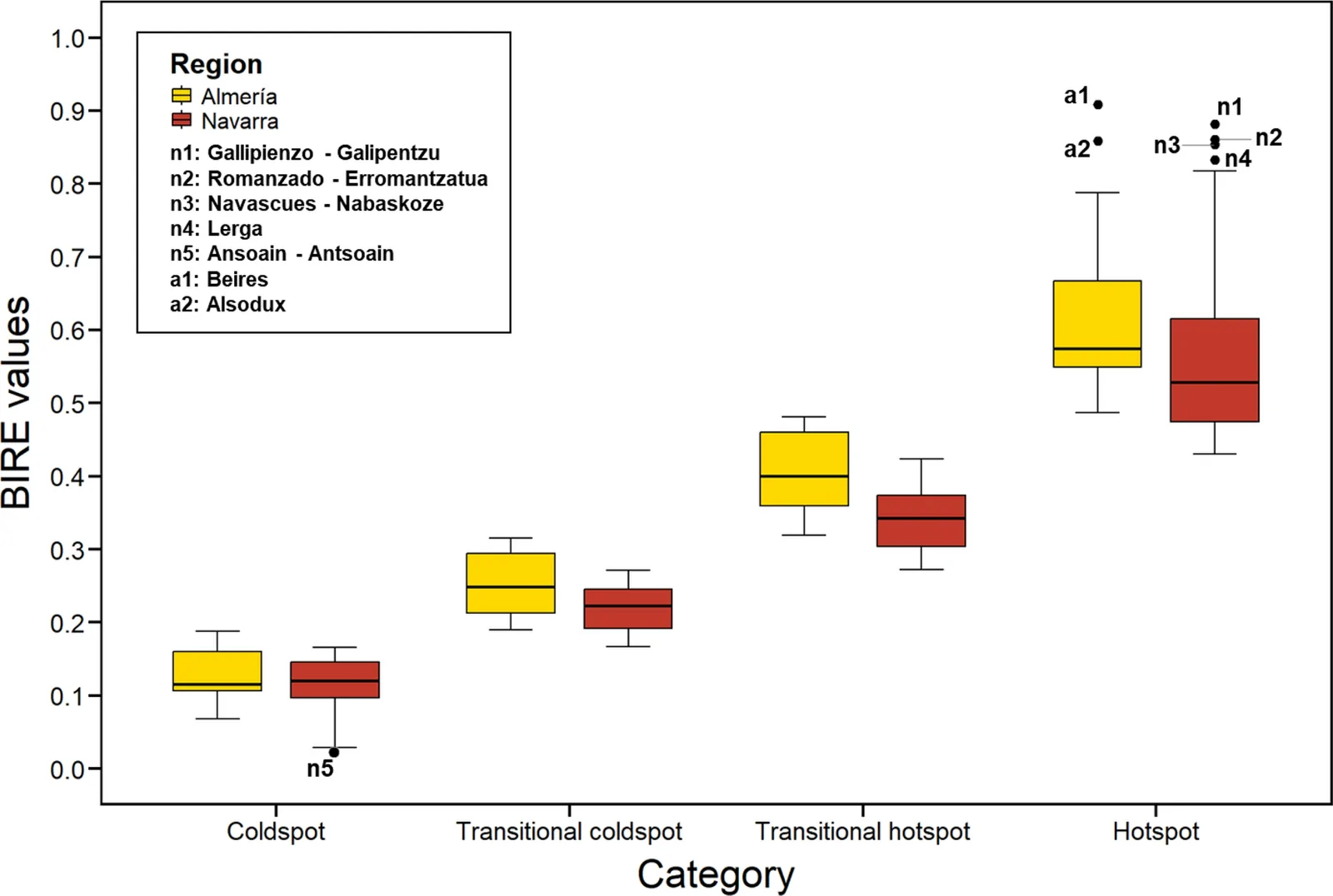
Distribution of BIRE. Boxplot representation of BIRE values across the four identified categories: coldspots, transitional coldspots, transitional hotspots, and hotspots, for both case studies (Almería in yellow and Navarra in red). The boxes represent the interquartile range (IQR), with the horizontal black line indicating the median. Whiskers extend to the smallest and largest values within 1.5 times the IQR, while dots indicate outliers. Specific municipalities with extreme values are labeled for reference (a: Almería and n: Navarra)

Differences emerged between regions in the relative positioning of municipalities across the four coldspot–hotspot categories (Fig. 4). Overall, BIRE values increased from coldspots to hotspots, reflecting a structured gradient in biocultural diversity. However, the dispersion of values within each category differed between Navarra and Almería. Navarra exhibited a broader spread of BIRE values, with a more pronounced variability across all categories, including both municipalities with exceptionally high values and others with notably low values. In contrast, Almería’s values tended to be more concentrated around the median, with fewer extreme cases in each category. Examination of outlier municipalities in Navarra confirmed that their markedly high scores across multiple variables represent genuine biocultural richness rather than artefacts of statistical normalization.

In both case studies (Fig. 4), coldspots (Z-score ≤ Q1) included municipalities with consistently low BIRE values across the three biocultural dimensions (i.e., materialized, lived, and stewardship) with particularly low scores in the stewardship dimension in most cases. In Navarra, one municipality (‘Ansoain–Antsoain’) emerged as an extreme low BIRE value outlier.

Transitional coldspots (Q1 < Z-score ≤ Q2) represented municipalities with moderately low BIRE values, resulting from moderate materialized and lived scores and consistently lower stewardship values. These municipalities did not exhibit extreme low biocultural diversity but showed a trend toward biocultural depletion.

Transitional hotspots (Q2 < Z-score ≤ Q3) were municipalities with moderate enrichment in biocultural diversity according to their BIRE values. They were characterized by higher materialized and lived scores, together with more heterogeneous stewardship values.

Hotspots (Z-score > Q3) represented municipalities with the highest BIRE values across the three dimensions. These areas consistently presented a high concentration of components in the materialized, lived, and stewardship dimensions. In both Navarra and Almería, hotspots were predominantly located in rural and mountainous municipalities. Beyond these general trends, the presence of outliers in the hotspots category highlighted key differences in the biocultural diversity patterns of each case study (Fig. 4). In the central-western part of Almería, ‘Beires,’ and ‘Alsodux’ emerged as the only significant outliers, whereas in Navarra, multiple municipalities in the central and northeast (‘Gallipienzo-Gallipientzu,’ ‘Romanzado-Erromantzatua,’ ‘Navascues-Nabaskoze,’ and ‘Lerga’) showed exceptionally high BIRE values, further emphasizing Navarra’s broader variability. The clustering of outliers in Almería suggested that high biocultural diversity in this province was more localized, whereas in Navarra, it was more dispersed across multiple municipalities. Nevertheless, Almería’s outliers shared certain characteristics with the outliers in Navarra, such as their rural character (as reflected by geographic and demographic variables in Appendixes S3 and S11); their proximity to mountainous areas; and the strong presence of traditional agricultural systems (as captured by the traditional agriculture variable in Table 1).

**Fig. 5:**
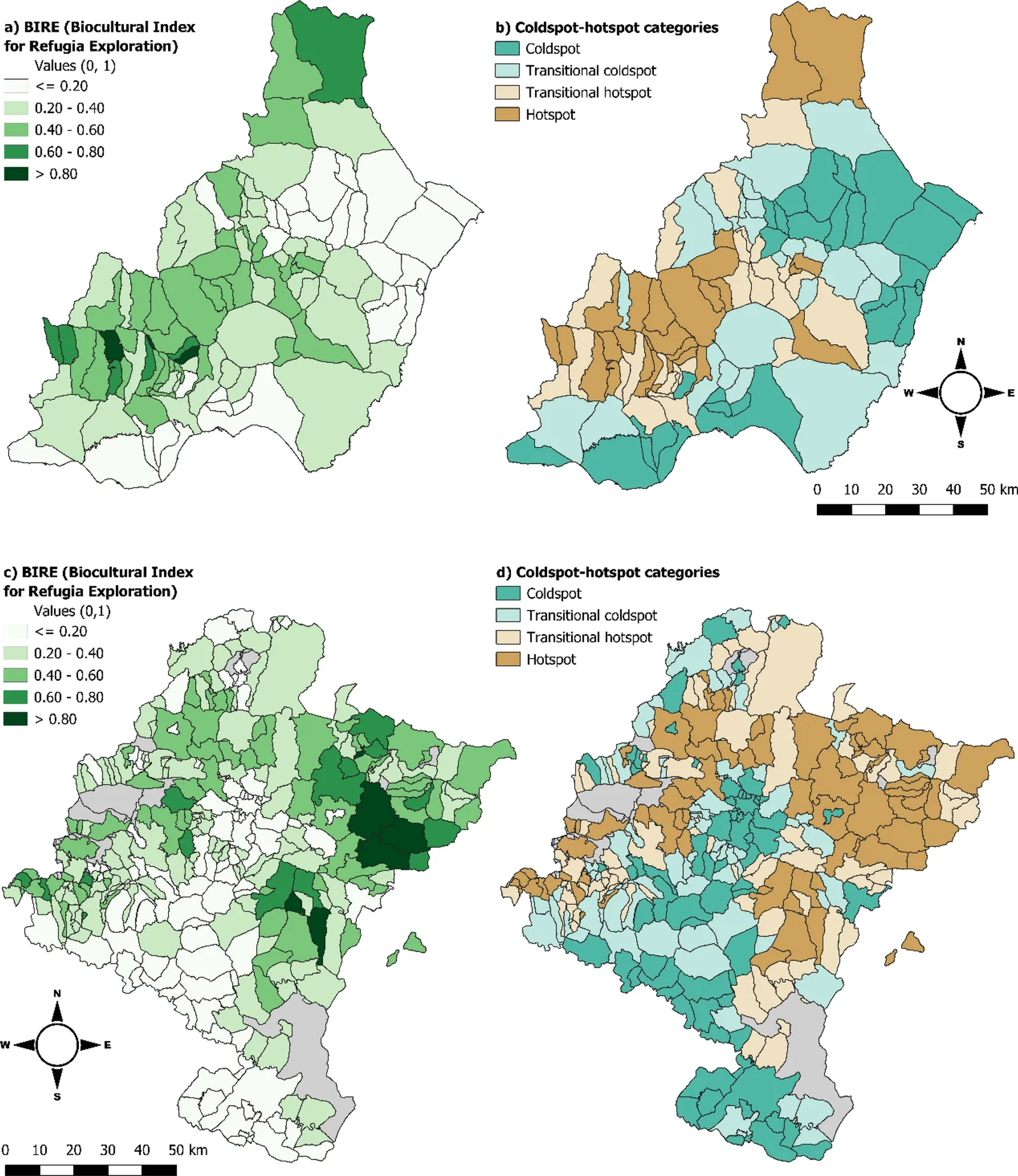
Maps of BIRE spatial distribution and coldspot–hotspot categories for municipalities in Almería (**a**, **b**) and Navarra (**c**, **d**). In Navarra, facerías (gray areas) were excluded, as they are not municipalities and lack census data for population normalization

BIRE values showed distinct spatial patterns across the two case studies (Fig. 5). In Almería (Fig. 5a), the highest values of BIRE (> 0.80) were concentrated in the southwestern, central, and northern parts of the province, those corresponding with the main hotspots associated with mountainous and rural areas (Fig. 5b). On the other hand, the lowest BIRE values (< 0.20), corresponding to coldspots, were observed in municipalities located in the southern and northeastern parts of the study area, which in these cases overlapped spatially with coastal zones and urban centers (Fig. 5b).

In Navarra (Fig. 5c), areas with high BIRE values were mainly concentrated in the northeast and eastern areas, where some municipalities exceeded 0.80. In contrast, municipalities in southern and southwestern Navarra generally exhibited lower index values. The highest BIRE values were predominantly observed in hotspot municipalities located in mountainous rural areas in the northern, eastern, and western regions, while the lowest values corresponded to coldspot municipalities concentrated in areas near cities or in municipalities characterized by flat topography and large populations (as reflected by geographic and demographic variables in Appendixes S3 and S11) in the southern and central regions (Fig. 5d).

The intermediate categories, transitional coldspots and transitional hotspots, were scattered in both case studies, with no clear concentration in specific areas. However, their long-term trajectory might be influenced by surrounding municipalities’ categories. For full details on the exact BIRE and Z-score values for each municipality, see Appendix S7.

**Fig. 6:**
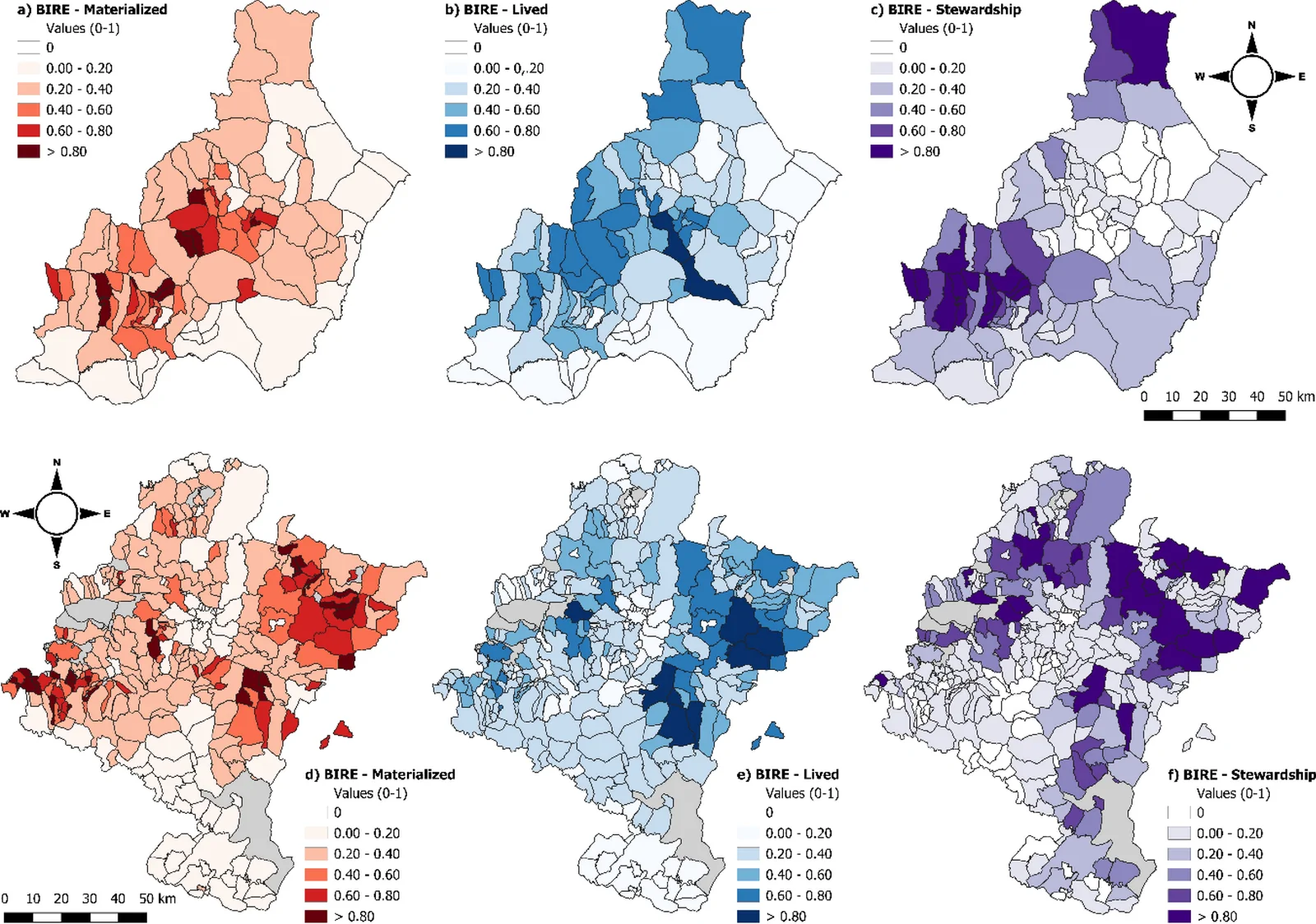
Maps of the materialized, lived, and stewardship dimensions of BIRE for Almería (**a**–**c**) and Navarra (**d**–**f**). In Navarra, facerías (gray areas) were not analyzed

The spatial distribution of the three biocultural dimensions of BIRE in Almería and Navarra is displayed in Fig. 6. In Almería, the materialized BIRE (Fig. 6a) had its highest values concentrated in the central and southwestern municipalities, where land use is predominantly characterized by protected mountainous areas. The lived BIRE’s (Fig. 6b) highest values were found in rural municipalities where agriculture is the dominant land use. The stewardship BIRE (Fig. 6c) highlighted some of Almería’s northern and western municipalities, particularly those located in Sierra Nevada and Sierra de María, comprising protected forested areas.

In Navarra, similar spatial patterns were observed. The materialized BIRE (Fig. 6d) had its highest values in northeastern and western municipalities, primarily in areas with large natural forested and mountainous landscapes and minimal urbanization. The lived BIRE (Fig. 6e) followed a distinct pattern, with elevated values primarily located in western, eastern, and northeastern municipalities, where agricultural lands coexist with forests, hunting areas, and livestock farming. The stewardship BIRE (Fig. 6f) had high values predominantly in the northern and northeastern municipalities, where the largest protected areas and extensive forested lands are concentrated.

## Discussion

This study introduces the Biocultural Index for Refugia Exploration (BIRE) as a novel, scalable tool for quantifying and mapping biocultural diversity at the municipal level. Our findings demonstrate the applicability of BIRE and reveal different patterns of values depending on land management types, rural-urban gradient and type of land tenure. The classification into coldspots, transitional coldspots, transitional hotspots, and hotspots highlights distinct social-ecological dynamics, while the analysis of materialized, lived, and stewardship dimensions confirmed the uneven spatial distribution of biocultural diversity components. Overall, BIRE provides an operational framework that can inform sustainable management, landscape planning, conservation prioritization, and policy design by linking spatial data with decision-making needs.

### Advancing the quantification of biocultural diversity

Quantifying biocultural diversity remains a significant challenge due to its complex, multidimensional, and context-dependent nature (Bridgewater and Rotherham 2019). Unlike biodiversity, which relies on well-established ecological metrics such as species richness or the Shannon index, biocultural diversity encompasses both tangible and intangible components, making systematic measurement difficult (Maffi 2007; Bridgewater and Rotherham 2019). Recently, a growing body of the literature has sought to adapt ecological diversity metrics to assess biocultural diversity. A notable example is Mateus-Aguilar et al. (2025), who applied Shannon and Simpson indices across multiple scales to evaluate biocultural diversity patterns in Colombia. While these adaptations offer promising advances, they still rely on metrics originally designed for ecological contexts that may not fully capture the multidimensional nature of human-nature relationships. Such relationships are shaped not only by material uses of biodiversity, but also by symbolic, spiritual, and identity-based connections that are deeply embedded in traditional societies (Diamond 2013). Consequently, there is still a need to develop locally relevant indicators specifically tailored to biocultural diversity, ensuring that both cultural and ecological realms and tangible and intangible components are adequately represented (Loh and Harmon 2005; Gonçalves et al. 2021).

One of the earliest large-scale efforts designed for this purpose was the Global Index of Biocultural Diversity (GBCD) proposed by Loh and Harmon (2005). This index integrated linguistic, religious, ethnic, animal, and plant diversities at the national level, offering valuable insights into global patterns of biocultural diversity. However, its broad spatial resolution limited its ability to reflect regional and local social-ecological dynamics. Since then, alternative approaches have emerged, including participatory mapping, ethnobotanical inventories, spatial models, and GIS-based analyses, aiming to bridge the gap between global-scale indicators and locally relevant assessment exercises. Building on this foundation, studies have refined methods to measure biocultural diversity at regional and local level. For instance, Gottero and Cassatella (2017) developed a biocultural diversity index for Italy’s Piedmont region, integrating ethnolinguistic diversity, certified agricultural products, traditional landscape features, and species richness to inform rural development policies. Similarly, Dobrovodská et al. (2019) applied a plot-based methodology in Slovakia, assessing species richness, rare species presence, and land-use practices to evaluate the biocultural value of traditional agricultural landscapes. In urban settings, several authors proposed indicator-based methodologies to integrate local ecological knowledge, community governance, and visitation rates for green infrastructure planning (Gonçalves et al. 2021). However, many of these indices have been designed for specific cultural or ecological settings, meaning that some of their indicators may not be relevant or applicable in other contexts. This highlights the need for approaches that can be tailored to the specific realities of each territory, while still following a consistent methodological pathway.

The Biocultural Index for Refugia Exploration (BIRE) was designed to address this need by providing a spatially explicit, multidimensional, and scalable tool for mapping biocultural diversity, while also being adaptable to different regions. By trying to bridge conceptual frameworks with operational tools, BIRE represents an effort to systematically quantify and map biocultural diversity in a way that is transferable to other social-ecological contexts. Instead of prescribing a universal set of indicators, BIRE offers a flexible structure in which locally relevant variables can be selected to best represent the ecological, cultural, and governance realms, organized into three complementary dimensions: materialized, lived, and stewardship (Elands et al. 2019). This design allows BIRE to accommodate different numbers of variables making it replicable across diverse geographical contexts and landscapes with minimal methodological adjustments. In contrast with the previous biocultural indices developed for highly specific settings, BIRE seeks to balance analytical transferability with contextual relevance while maintaining flexibility in the selection of variables.

Beyond methodological advancements, BIRE shows strong potential as both a diagnostic tool and a decision-support system for policymakers in conservation, landscape planning, sustainable management, and social-ecological research. As data availability improves and locally grounded variables are refined or expanded, BIRE could be integrated into conservation planning frameworks and tools such as MARXAN (Ball et al. 2009), allowing biodiversity conservation and cultural heritage protection to reinforce one another. At a regional level, BIRE can help identify biocultural refugia and map areas of risk or opportunity—detecting municipalities where biocultural diversity is declining or where biocultural potential remains untapped. This capability makes it a valuable tool for early intervention, enabling policymakers to implement targeted research, conservation measures, or restoration strategies before critical components of biocultural diversity disappear. Moreover, by providing a structured, data-driven approach, BIRE contributes to discussions on Indigenous and community-led conservation governance, recognizing the pivotal role of local communities in sustaining biocultural landscapes (Maffi 2007).

### Spatial patterns of BIRE and their implications for conservation and management in Almería and Navarra

Analyzing the spatial patterns of BIRE in Almería and Navarra provides insights into how different land-use contexts and social-ecological dynamics shape opportunities and constraints for conservation and management. It is important to note that the results from Almería and Navarra are not directly comparable in absolute terms, as the variable sets differ to reflect the specific ecological and cultural realities of each territory. Rather than enabling direct interregional comparison, BIRE was applied to explore within-region spatial patterns and to test whether the index performs consistently across contrasting social-ecological contexts while maintaining methodological coherence and adapting to local realities.

The application of BIRE in both Almería and Navarra revealed clear spatial patterns of biocultural diversity: higher values in rural and mountainous areas, and lower values in urban and highly transformed locations. These results align with previous studies highlighting the role of traditional agricultural landscapes (Agnoletti et al. 2015; Quintas-Soriano et al. 2023), as well as communal land management, and protected areas in sustaining biocultural richness (Barthel et al. 2013; Ferrara et al. 2020; Otamendi-Urroz et al. 2025). In Almería, hotspots and transitional hotspots were concentrated in the southwestern and northern mountains, where protected areas, forests, and traditional agro-pastoral systems persist (Quintas-Soriano et al. 2016, 2019) although they remain under the pressures of rural abandonment and depopulation (Prus et al. 2022; López-Zayas et al. 2024). By contrast, coastal and peri-urban or urban municipalities showed the lowest values due to tourism, greenhouse agriculture, quarrying, and demographic change (Quintas-Soriano et al. 2016; Castro et al. 2019; Otamendi-Urroz et al. 2023). A similar pattern was observed in Navarra, with hotspots clustered in the Pyrenees and other eastern and western mountain regions characterized by extensive livestock farming and communal land tenure and an ongoing depopulation risk (Labiano-Mangado 2021; Albizua et al. 2024). Coldspots were mainly located around cities such as Pamplona, Tudela, and their surroundings, where high population density and modern lifestyles combined with urbanization and industrialization (Labiano-Mangado 2021) erode traditional agricultural and communal practices (Zaratiegui 1988; Prus et al. 2022). However, lower values in urban areas should be interpreted cautiously, as cities can also host distinct forms of biocultural diversity (e.g., urban biodiversity, diverse cultural identities, hybrid knowledge systems, and novel human-nature interactions) (Elands et al. 2019) that might not be fully captured by the variables used in this study.

In general, broader demographic dynamics emerge as a key driver shaping biocultural patterns across both regions. Our results highlight a risk for many municipalities: on the one hand, complete depopulation threatens the survival of biocultural knowledge and practices, while on the other, excessive population growth and urban expansion can erode biocultural heritage through the replacement of traditional systems with urban lifestyles disconnected from nature (Pretty et al. 2009). This suggests that biocultural refugia may be more likely to emerge and persist within an optimal demographic range. Furthermore, recent work has conceptualized biocultural refugia as existing along a continuum from relatively isolated systems to highly dynamic and human-influenced landscapes (Otamendi-Urroz et al. 2025). In this context, the transitional categories identified by BIRE help capture these intermediate configurations where biocultural components persist under varying degrees of pressure. BIRE also provides insights into the potential trajectories of these transitional hotspots and coldspots, as their long-term trajectory might be influenced by the surrounding territorial context. For instance, a transitional coldspot enclosed by other coldspots is more likely to experience further biocultural erosion. In contrast, transitional hotspots, even when surrounded by multiple coldspots, may hold added value as relative biocultural refugia along the continuum, as they retain components of biocultural diversity despite strong external pressures. These municipalities could evolve into full hotspots if favorable ecological, cultural, or policy-related conditions support the persistence and accumulation of biocultural components; however, if their biocultural value is overlooked or not actively maintained, they may instead decline toward colder categories.

Within this dynamic perspective, hotspots—where traditional knowledge, cultural practices, and environmental features converge—particularly municipalities classified as outliers, acquire particular relevance not only as areas of high biocultural value, but also as biocultural refugia and potential reference systems for guiding management and conservation. As an example, municipalities with strong communal governance, diversified traditional agricultural practices, or well-established cultural heritage policies could offer strategic guidance for areas aiming to strengthen their biocultural diversity. By examining what distinguishes these hotspots from their surroundings, local decision-makers can develop targeted interventions to reinforce biocultural diversity in transitional hotspots. Additionally, these insights can guide the restoration of coldspots, helping to reintroduce degraded biocultural components affected by urbanization, agricultural intensification, or demographic shifts (Prus et al. 2022; Quintas-Soriano et al. 2023). Moreover, BIRE can help anticipate changes in transitional coldspots, enabling early interventions to prevent further biocultural erosion before it becomes irreversible.

Beyond spatial categorization into hotspots and coldspots, examining the internal structure of BIRE and its three dimensions (i.e., materialized, lived, and stewardship) provides additional insights by allowing each component of biocultural diversity to be mapped and interpreted separately. This dimensional disaggregation makes it possible to identify specific areas for improvement within each domain. Growing evidence shows that human cultures actively shape and sustain biodiversity through long-term management practices and stewardship systems (Levis et al. 2024), highlighting the critical role of governance, institutions, and collective action in maintaining biocultural diversity. In this context, a municipality with high materialized diversity but low stewardship diversity may benefit from management actions and conservation policies that promote communal governance, participatory approaches, or locally grounded stewardship programs. Similarly, municipalities with low lived diversity may require initiatives aimed at revitalizing traditional knowledge systems, cultural festivals, or forms of ecological tourism rooted in local practices. This level of specificity enables more targeted and effective policy interventions at the municipal scale, ensuring that management and conservation efforts are adapted to the unique social-ecological realities of each case study.

### Limitations

This study has several limitations that must be acknowledged. The most significant constraint is the availability of spatial data, particularly for intangible cultural components. These cultural dimensions are not systematically recorded in existing databases, making it necessary to rely on quantitative proxy variables derived from secondary data sources such as remote sensing records that may not fully capture their complexity (Bridgewater and Rotherham 2019). Future efforts could expand data collection through socio-cultural approaches such as qualitative interviews, participatory mapping, and community-based surveys that more accurately capture perceptions, values, practices, and knowledge transmission processes. When appropriately designed, these approaches can be translated into spatially explicit or mixed-method indicators without reducing the richness of lived cultural relationships to overly reductive or decontextualized metrics.

Differences in variable availability between case studies were also a limitation. Certain variables were available for one region but not for the other, constraining exact equivalence between Almería and Navarra. While the selected variables were conceptually aligned within the three dimensions (i.e., materialized, lived, stewardship), proxy variables had to be used in some cases. For instance, open-air agricultural area was used as a proxy for traditional agriculture, defined here in contrast with greenhouse-based systems, as available national spatial datasets do not directly distinguish between different management intensities or production systems within specific crop types (e.g., extensive versus intensive olive groves). This not only affects the degree of comparability between regions, but also constrains the interpretation of results, as some proxies capture broad spatial patterns rather than specific practices, experiences, or management regimes. Nonetheless, the consistent application of the three-domain structure and the use of percentile-based scoring mitigate this issue by ensuring that the index remains coherent and interpretable within each region.

Furthermore, the unequal number of variables used across the three biocultural dimensions could have introduced weighting imbalances. However, sensitivity analyses indicated that the index remains robust even when dimensions contain different numbers of variables. Importantly, BIRE accounts for such asymmetries by weighting scores by the number of nonzero variables and by averaging across dimensions.

Finally, the interpretation of low biocultural diversity values in urban and peri-urban areas should also be considered with caution. Although urbanization can contribute to the erosion of certain traditional practices and ecological components, cities may also host diverse cultural expressions and forms of biocultural diversity (Elands et al. 2019) that might not be fully captured by the variables used in this study. As such, observed coldspots may partly reflect the types of variables available and selected, highlighting how different dimensions of urban biocultural diversity can remain underrepresented rather than absent. Future applications of BIRE could incorporate urban-specific indicators to better account for urban biocultural diversity and avoid underrepresentation of socio-cultural and ecological dynamics in highly transformed environments.

Despite these limitations, BIRE remains a replicable and adaptable tool for mapping biocultural diversity. Nevertheless, addressing these limitations through improved data collection, continued methodological refinement, and balanced dimensional representation is still necessary to strengthen BIRE’s applicability across diverse social–ecological contexts.

## Conclusion

This study presents the Biocultural Index for Refugia Exploration (BIRE) as a novel, scalable, and transferable approach for mapping biocultural diversity across different contexts. By integrating diverse biological and cultural indicators, BIRE provides a spatially explicit tool that enables the identification of biocultural hotspots, transitional hotspots, coldspots, and transitional coldspots, with the potential to offer valuable insights for sustainable management, conservation, and policy development.

The application of BIRE to Navarra and Almería shows its capacity to reveal distinct spatial patterns of biocultural diversity. While rural and mountainous municipalities consistently exhibit higher BIRE values, urbanized areas show lower scores, reflecting the pressures of land-use change, demographic shifts, and the erosion of traditional knowledge. Beyond its analytical capabilities, BIRE could serve as a practical tool for sustainable management in these areas, supporting decision-making processes by identifying priority areas for biocultural conservation and restoration.

Despite its limitations, BIRE offers a replicable and actionable methodology that can bridge the gap between biocultural theory and practical metrics. Its effectiveness ultimately depends on the availability, quality, and spatial resolution of the underlying data, which may require context-specific adaptations to ensure robust and meaningful assessments. At the same time, the use of publicly available spatial datasets and a flexible proxy-based structure enhances BIRE’s scalability, enabling its application at regional, national, or global scales where comparable data sources exist. Future research should therefore focus on incorporating additional intangible cultural variables and testing the performance of BIRE across different governance scales.

## Supplementary Information

Below is the link to the electronic supplementary material.

Supplementary file1 (PDF 606 KB)

## Data Availability

All data supporting the findings of this study are openly available. The complete set of variables, intermediate results, maps, statistical outputs, and scripts used to compute the Biocultural Index for Refugia Exploration (BIRE) are provided in the Supplementary Information as Figshare links. Any additional raw or intermediate data not included in these repositories are available from the corresponding author upon reasonable request.

## Footnotes

1 https://geoportal.navarra.es/es/idena.

2 https://portalrediam.cica.es/descargas.

3 https://qgis.org/.
