# Supplementary material for: Mapping biocultural diversity: An integrative spatial approach to inform conservation and management decisions
Source: Ambio. 2026 Mar 14;55(10):2480–97. doi: 10.1007/s13280-026-02370-6 (PMC13553999; doi:10.1007/s13280-026-02370-6)
Supplement: Supplementary file 1 [file 13280_2026_2370_MOESM1_ESM.pdf]

Mapping Biocultural Diversity: an integrative spatial approach to inform conservation and management decisions

*** Supplementary material may be found in the online version of the article at the publisher's website.*

SUPPLEMENTARY INFORMATION

Data is provided within supplementary information files:

Appendix S1. List of all variables. https://figshare.com/s/d3391e6695fd91cba85d

Appendix S2. Biocultural diversity variables per municipality. https://figshare.com/s/526c21ca3645a131dba8

Appendix S3. Explanatory variables per municipality. https://figshare.com/s/ff99e321a0a6bd9210c4

Appendix S4. Maps of all biocultural diversity variables in Navarra. https://figshare.com/s/4e72065e8a2894989661

Appendix S5. Maps of all biocultural diversity variables in Almería. https://figshare.com/s/db1871296cb05af03c55

Appendix S6. Index calculation example. https://figshare.com/s/4f17bd2e5db20393a94c

Appendix S7. Index results per municipality. https://figshare.com/s/4387c3980fbb14e33d6f

Appendix S8. Index distribution.

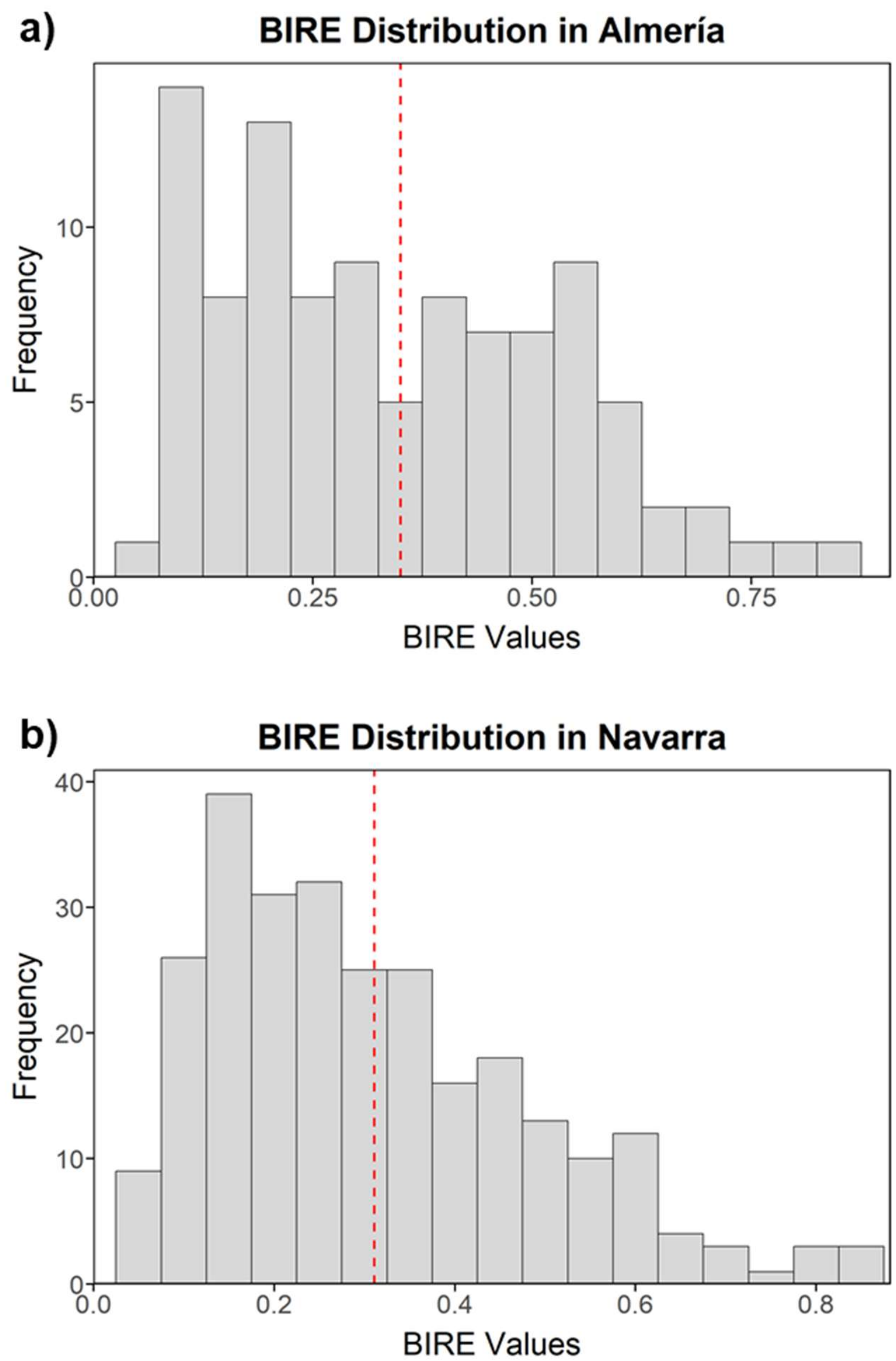

Fig. S8.1. *Empirical distribution of the Biocultural Index for Refugia Exploration (BIRE) in Almería (a) and Navarra (b). The red dashed line indicates the provincial mean BIRE value in each case.*

Appendix S9. Bootstrap results. https://figshare.com/s/8139e64aa5e1e8b14056

Appendix S10. LOVO and LODO results. https://figshare.com/s/3c76734de61ae5d9318f

Appendix S11. Variance partitioning. https://figshare.com/s/850d3505813e6fbd10f8
